# TpTe and TpTe/QT: novel markers to predict sudden cardiac death in ESRD?

**DOI:** 10.1590/2175-8239-JBN-2017-0021

**Published:** 2018-08-16

**Authors:** Basil M. Saour, Jeffrey H. Wang, Michael P. Lavelle, Roy O. Mathew, Mandeep S. Sidhu, William E. Boden, Joseph D. Sacco, Eric J. Costanzo, Mohammad A. Hossain, Tuhsar Vachharanji, Anas Alrefaee, Arif Asif

**Affiliations:** 1 Albany Medical College, Albany, NY, USA.; 2 Stratton VA Medical Center, Department of Medicine, Division of Cardiology, Albany, NY, USA.; 3 Albany Medical College, Department of Medicine, Division of Cardiology, Albany, NY, USA.; 4 Hennepin County Medical Center, Department of Medicine, Division of Nephrology, Minneapolis, MN, USA.; 5 WJB Dorn VA Medical Center, Department of Medicine, Division of Nephrology, Columbia, SC, USA.; 6 Jersey Shore University Medical College, Seton Hall Hackensack-Meridian School of Medicine, Department of Medicine, Neptune, New Jersey, USA.; 7 Salisbury VA Health Care System, Department of Nephrology, North Carolina, USA.

**Keywords:** Death, Sudden, Kidney Failure, Chronic, TpTe, Morte Súbita, Falência Renal Crônica, TpTe

## Abstract

**Introduction:**

Reliable markers to predict sudden cardiac death (SCD) in patients with end stage renal disease (ESRD) remain elusive, but echocardiogram (ECG) parameters may help stratify patients. Given their roles as markers for myocardial dispersion especially in high risk populations such as those with Brugada syndrome, we hypothesized that the Tpeak to Tend (TpTe) interval and TpTe/QT are independent risk factors for SCD in ESRD.

**Methods:**

Retrospective chart review was conducted on a cohort of patients with ESRD starting hemodialysis. Patients were US veterans who utilized the Veterans Affairs medical centers for health care. Average age of all participants was 66 years and the majority were males, consistent with a US veteran population. ECGs that were performed within 18 months of dialysis initiation were manually evaluated for TpTe and TpTe/QT. The primary outcomes were SCD and all-cause mortality, and these were assessed up to 5 years following dialysis initiation.

**Results:**

After exclusion criteria, 205 patients were identified, of whom 94 had a prolonged TpTe, and 61 had a prolonged TpTe/QT interval (not mutually exclusive). Overall mortality was 70.2% at 5 years and SCD was 15.2%. No significant difference was observed in the primary outcomes when examining TpTe (SCD: prolonged 16.0% *vs*. normal 14.4%, *p*=0.73; all-cause mortality: prolonged 55.3% *vs*. normal 47.7%, *p*=0.43). Likewise, no significant difference was found for TpTe/QT (SCD: prolonged 15.4% *vs*. normal 15.0%, *p*=0.51; all-cause mortality: prolonged 80.7% *vs*. normal 66.7%, *p*=0.39).

**Conclusions:**

In ESRD patients on hemodialysis, prolonged TpTe or TpTe/QT was not associated with a significant increase in SCD or all-cause mortality.

## RESUMO

**Introdução:**

Marcadores confiáveis para predizer morte súbita cardíaca (MSC) em pacientes com doença renal terminal (DRT) permanecem elusivos, mas os parâmetros do ecocardiograma (ECG) podem ajudar a estratificar os pacientes. Devido a seus papéis como marcadores para a dispersão miocárdica, especialmente em populações de alto risco, como aquelas com síndrome de Brugada, nós hipotetizamos que o intervalo pico da onda T ao final da onda T (TpTe) e TpTe/QT são fatores de risco independentes para MSC na DRT.

**Métodos:**

Revisão retrospectiva do prontuário foi realizada em uma coorte de pacientes com DRT iniciando a hemodiálise. Os pacientes eram veteranos de guerra americanos que utilizavam os centros médicos do Veterans Affairs para atendimento médico. A idade média de todos os participantes foi de 66 anos e a maioria era do sexo masculino, consistente com uma população veterana dos EUA. ECGs que foram realizados dentro de 18 meses após o início da diálise, e foram avaliados manualmente para TpTe e TpTe/QT. Os desfechos primários foram MSC e mortalidade por todas as causas, e estes foram avaliados até 5 anos após o início da diálise.

**Resultados:**

Após o critério de exclusão, foram identificados 205 pacientes, dos quais 94 com TpTe prolongado e 61 com intervalo TpTe/QT prolongado (não mutuamente exclusivo). A mortalidade geral foi de 70,2% em 5 anos e a MSC foi de 15,2%. Nenhuma diferença significativa foi observada nos desfechos primários ao se avaliar o TpTe (MSC: prolongado 16,0% versus normal 14,4%, *p* = 0,73; mortalidade por todas as causas: prolongado 55,3% *vs*. normal 47,7%, *p* = 0,43). Da mesma forma, nenhuma diferença significativa foi encontrada para TpTe/QT (MSC: prolongado 15,4% *vs*. normal 15,0%, *p* = 0,51; mortalidade por todas as causas: prolongado 80,7% *vs*. normal 66,7%, *p* = 0,39).

**Conclusões:**

Em pacientes com insuficiência renal terminal em hemodiálise, TpTe ou TpTe/QT prolongados não foram associados a um aumento significativo da morte súbita ou mortalidade por todas as causas.

## INTRODUCTION

Sudden cardiac death (SCD) is the leading cause of mortality in patients with end stage renal disease (ESRD) treated with hemodialysis, accounting for 26.9% of all deaths in this population.^1^ In the United States, the incidence of SCD in the general population is 53/100,000; the primary identifiable risk factors are reduced systolic function with a depressed left ventricular ejection fraction or a history of prior sudden cardiac arrest.^2^^-^ 4 These characteristics do not have the same predictive ability in ESRD. Bleyer et al. reported that 75% of dialysis patients who died of SCD have a left ventricular ejection fraction > 35%.^5^ To date, there is no reliable risk stratification marker to identify dialysis patients at high arrhythmic risk for sudden cardiac arrest.^5^^-^ 7

Current efforts aimed at identifying SCD risk stratification markers have focused on ECG data. In the general population, ECG findings with validated evidence to support primary prevention of SCD with an implantable cardioverter-defibrillator (ICD) are those linked to an underlying cardiomyopathy or impaired ion channel function, such as Brugada pattern, Arrhythmogenic Right Ventricular Dysplasia with an Epsilon wave, or prolonged QT.^8^ Electrolyte and fluid shifts during hemodialysis combined with the increased prevalence of myofibrosis in this population, is thought to predispose individuals to ventricular arrhythmias, which may manifest as derangements in ECG parameters.^6^^,^ 9 Recent literature suggests that various ECG changes, such as prolonged PR, QRS, or QTc intervals, may be independent risk predictors for cardiovascular (CV) death in patients with chronic kidney disease.^10^^-^ 12

Tpeak-Tend (TpTe) and TpTe/QT intervals are ECG markers of arrhythmogenesis, which reflect the degree of heterogeneity of repolarization in the myocardium.^13^ In the general population, a prolonged TpTe is associated with a 2-fold higher risk of SCD.^14^ Furthermore, prolonged TpTe or prolonged TpTe/QT intervals have demonstrated potential utility for prediction of SCD in patients with hypertrophic obstructive cardiomyopathy, long QT syndrome, and those undergoing percutaneous coronary intervention.^15^^,^ 16 Although hemodialysis has been shown to prolong the TpTe interval, no study have examined the predictive ability of a baseline TpTe interval in patients with ESRD.^17^ We hypothesized that TpTe and TpTe/QT are independent risk factors for SCD in ESRD. The aim of this study was to assess the prognostic value of TpTe and TpTe/QT for SCD in ESRD patients, independent of the mechanism for prolongation of the TpTe interval.

## METHODS

### STUDY POPULATION

This retrospective cohort study included veterans with ESRD from the 5 upstate New York Veterans Affairs medical centers. All data was obtained from clinical information that was already collected and stored within the Veterans Affairs corporate data warehouse, no patients were formally interviewed or examined as the study was retrospective in nature. All consecutive patients who initiated outpatient in-center hemodialysis between January 1, 2000, and December 31, 2007, and dialyzed for at least 90 days were included.

Although it is routine that an ECG be conducted prior to the initiation of hemodialysis, this was unfortunately not always done in our patient sample. Furthermore, given the dynamic nature of ECGs in patients, especially in those with advanced renal disease, the first ECG that was examined was often not interpretable for evaluation of the TpTe segment. Thus, we defined the "baseline" ECG as the first suitable ECG after dialysis initiation.

Inclusion criteria were age > 18 and having an ECG within 18 months of dialysis initiation. Exclusion criteria were patients not dialyzed within the study time-frame, missing dialysis initiation date, unsuitable ECG (not in sinus rhythm, poor technical quality, left bundle branch block, QRS > 120ms), pre-existing ICD or permanent pacemaker (PPM), pregnancy, renal transplantation, or treated with peritoneal or home hemodialysis.

The Albay Stratton VA Medical Center Institutional Review board and Research and Development Committee approved this study under expedited review.

### ELECTROCARDIOGRAPHIC ANALYSIS

**Figure 1:**
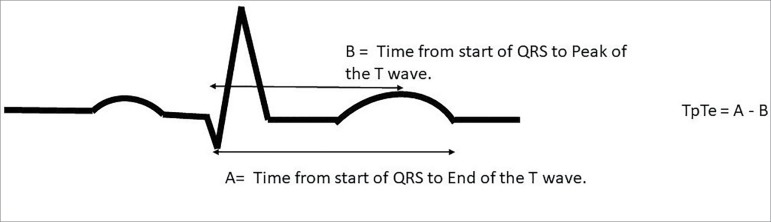
Pictorial representation illustrating how TpTe was calculated.

ECGs were analyzed at 25 mm/s paper speed and 10 mm/mV amplitude. All measurements were performed by a board-certified cardiologist. Baseline parameters from the ECG included manual measurements of the TpTe segment and QT interval. TpTe was calculated from the difference of the QT interval and the QRS complex to Tpeak interval (Figure 1). The QT interval was measured from the beginning of the QRS complex to the end of the T wave. The corrected QT interval (QTc) was obtained using Bazett's formula (QTc = QT/√RR interval). A prolonged TpTe segment was defined as > 85 ms, while a prolonged TpTe/QT segment was defined as > 0.25.^14^ The axis, presence of left ventricular hypertrophy (via Sokolow-Lyon criteria), right bundle branch block, non-specific intraventricular conduction delay, and left anterior or posterior fascicular block were recorded. QRS duration, heart rate, and PR interval were obtained from the ECG computer measurement. In accordance with previous studies, lead V5 was used for the measurements.^14^^-^ 16 If V5 was not interpretable, V4, then V6 was used.

### POWER ANALYSIS

There is no previous report in the literature on the rate of SCD in dialysis patients with a normal TpTe interval to guide the sample size calculation. Although we acknowledge that ESRD patients are a different substrate than the general population, using data from the general population we estimated that a dialysis patient with a normal TpTe has a 19% probability of dying from SCD 5 years after dialysis initiation.^14^ We hypothesized that having a prolonged TpTe interval increases the probability of SCD at 5 years by 2.2-fold. Thus, if we had 2 controls per case, we needed 135 patients (45 with prolonged TpTe and 90 with non-prolonged TpTe) to be able to reject the null hypothesis with power of 0.8. The type 1 error probability associated with this test of null hypothesis is 0.05. The sample size calculation was performed with the Power and Sample Size Program 3.0 (Vanderbilt University, Nashville, TN).

### ADJUDICATION OF SUDDEN CARDIAC DEATH

Mortality status and cause of death were obtained from the Centers for Medicare and Medicaid Services 2746 death notification form through a data request to the United States Renal Data System registry. Death from cardiac arrhythmia or cardiac arrest, cause unknown, was considered meeting criteria for SCD. Outcomes were assessed up to a maximum of 5 years following initiation of hemodialysis therapy.

### DATA ANALYSIS

Statistical analyses were conducted using SigmaPlot 12 (San Jose, CA). Baseline characteristics between subjects with a prolonged TpTe (and TpTe/QT) were compared using the two-tailed unpaired Student's t-test for continuous variables and the Pearson χ^2^ test for categorical data. Time-to-event analysis was performed using the Kaplan-Meier method with log-rank test. Statistical significance was defined as *p* < 0.05.

## RESULTS

### PATIENT SELECTION

**Figure 2:**
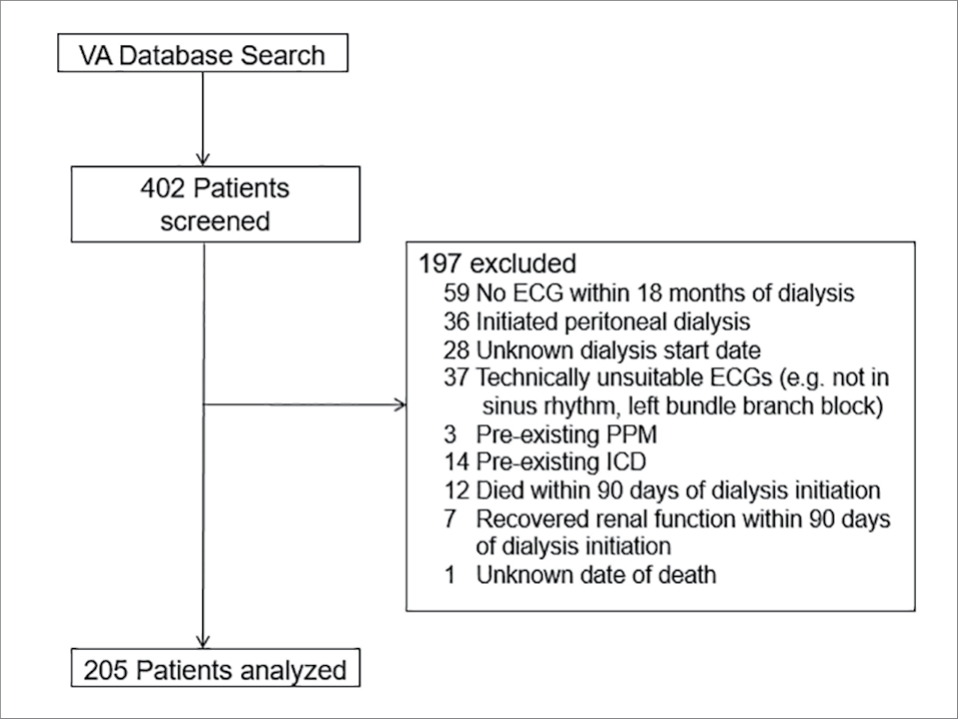
Patient selection.

The initial search of the VA database yielded 402 patients. After exclusion criteria were applied, 205 subjects remained. The major reasons for exclusion were the absence of an acceptable ECG within 18 months after starting dialysis (N = 59), use of peritoneal dialysis (N = 36), and technically unsuitable ECG (N = 37) (Figure 2). Of the 205 that remained, 94 were found to have a prolonged TpTe, while 61 had a prolonged TpTe/QT.

### BASELINE CHARACTERISTICS

Of the 205 identified patients, 99.5% were male, 66.8% were Caucasian and the mean age was 66.6 +/-12.3 years (Table 1). The mean duration on dialysis prior to the first ECG being obtained was 104 +/- 11.7 days.

**Table 1:** Baseline demographics. Data are reported as mean ± SD unless stated otherwise

	All (n = 205)	Normal TpTe (n = 111)	Prolonged TpTe (n = 94)	*p* value (Normal *vs.* Prolonged TpTe)	Normal TpTe/QT (n = 144)	Prolonged TpTe/QT (n = 61)	*p* value (Normal *vs*. Prolonged TpTe/QT)
Age, years	66.6 ± 12.3	65.9 ± 12.4	67.3 ± 12.2	0.40	65.7 + 1.1	67.5 + 1.5	0.36
Gender, males (%)	99.5	100	98.9	0.93	100	98.4	0.66
Time to ECG, days	104 ± 11.7	95 ± 11.9	115 ± 11.4	0.069	94 + 9.6	128 + 15.3	0.055
Race, n (%)							
White	137 (66.8)	66 (59.5)	71 (75.5)	0.022	93 (64.6)	44 (72.1)	0.58
African American	61 (29.8)	41 (36.9)	20 (21.3)	0.18	45 (31.3)	15 (24.6)	0.55
Cause of ESRD, n (%)							
Diabetic nephropathy	94 (45.9)	51 (45.9)	43 (45.7)		64 (44.4)	30 (49.2)	
Hypertension	24 (11.7)	15 (13.5)	9 (9.6)		18 (12.5)	6 (9.8)	
Glomerular disease	22 (10.7)	11 (9.9)	11 (11.7)		17 (11.8)	5 (8.2)	
Acute kidney injury	15 (7.3)	10 (9)	5 (5.3)		12 (8.3)	3 (5)	
Obstruction	8 (3.9)	2 (1.8)	6 (6.4)		4 (2.8)	4 (4.9)	
Ischemic nephropathy	5 (2.4)	5 (4.5)	0 (0)		5 (3.5)	0 (0)	
Polycystic kidney	5 (2.4)	3 (2.7)	2 (2.1)		3 (2.1)	2 (3.3)	
Unknown/Other	32 (15.6)	14 (12.6)	18 (19.2)		21 (14.6)	11 (18)	
ECG (mean, 95% CI)							
PR interval		170 (152, 194)	168 (151, 194)	0.99	172 (152,194)	166 (148,194)	0.47
QRS duration, ms		90 (84, 102)	98 (89.5, 112)	0.01	92 (84, 102)	98 (90, 114)	0.05
Left ventricularhypertrophy, n (%)		8 (7.2)	12 (12.8)	0.27	12 (8.3)	8 (13.1)	0.41
Fascicular Block		9 (8.1)	9 (9.6)	0.9	3 (8.1)	5 (8.2)	1
RBBB		12 (10.8)	13 (13.8)	0.66	3 (8.1)	5 (8.2)	1
QTc interval, ms		451 (426, 470)	453 (434, 476)	0.3	447 (426, 473)	457 (434, 475)	0.2
TpTe interval, ms		71.3 (56.3, 80.5)	103.5 (93.8, 120)	< 0.001	n/a	n/a	
TpTe/QTc interval, ms		n/a	n/a		0.18 (0.15, 0.21)	0.28 (0.26, 0.32)	< 0.001
Comorbidities n (%)							
Hypertension	182 (88.8)	97 (87.4)	85 (90.4)	0.64	128 (88.9)	54 (88.5)	0.87
Diabetes	135 (65.9)	74 (66.7)	61 (64.9)	0.91	96 (66.7)	39 (63.9)	0.83
CAD	87 (42.4)	46 (41.4)	41 (43.6)	0.86	63 (43.8)	24 (39.3)	0.67
CHF	80 (39.0)	37 (33.3)	43 (45.7)	0.09	54(37.5)	26 (42.6)	0.6
BMI (Mean, 95% CI)		27.4 (24.8, 32.3)	28.9 (24.3, 32.3)	0.54	28.2 (25.1, 32.3)	28.4 (23.9, 32)	0.75

BMI: body mass index; CAD: coronary artery disease; CHF: congestive heart failure; CI: confidence interval; ECG: electrocardiogram; ESRD: end stage renal disease; PR: PR interval; QTc: corrected QT interval; RBBB: right bundle branch block; TpTe: T peak to T end interval; TpTe/QT: TpTe interval corrected for the QT interval.

### NORMAL *VS*. PROLONGED TPTE

Caucasians were more likely to have prolonged TpTe intervals (66/137 [48%] normal *vs*. 71/137 [52%] prolonged, *p* = 0.022), whereas there was no statistically significant difference in numbers of African Americans with prolonged or normal TpTe or TpTe/QT intervals. There was no statistically significant difference in the proportion of patients with prolonged or normal TpTe with hypertension, congestive heart failure or other comorbidities (Table 1). The QRS duration was significantly longer in the prolonged TpTe group compared to normal TpTe (98 ms *vs*. 90 ms, *p* = 0.01). There was no statistical difference between normal or prolonged TpTe groups with regards to any other ECG parameter evaluated.

### NORMAL *VS*. PROLONGED TPTE/QT

There was no statistically significant difference between normal and prolonged TpTe/QT patients in any demographic or co-morbid condition (Table 1). In contrast to the TpTe comparisons, there was no difference in racial category distribution between prolonged TpTe/QT and normal TpTe/QT groups. The QRS duration was significantly longer in the prolonged TpTe/QT group (98 ms prolonged *vs*. 92 ms normal, *p* = 0.046) but no other ECG parameter was significantly different between the two groups.

### OUTCOMES - NORMAL *VS*. PROLONGED TPTE

**Figure 3:**
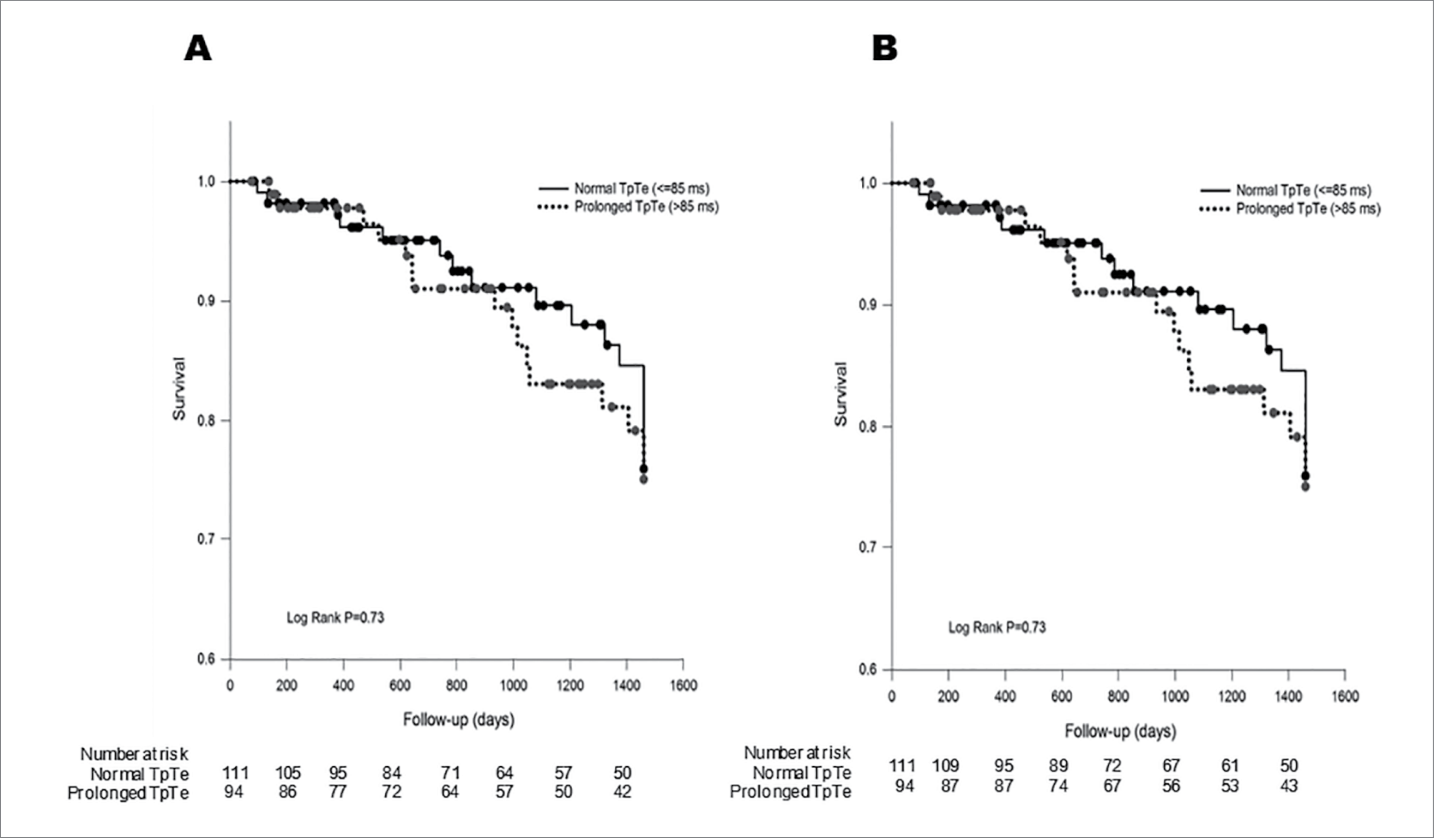
Overall survival (A) and survival without sudden cardiac death (B) between hemodialysis patients with normal and prolonged TpTe interval.

Subjects were followed for a mean of 3.5 years. The mean survival times for patients with a normal and prolonged TpTe interval after dialysis initiation were 2.91 and 2.83 years, respectively (Figure 3A). No significant difference was observed in the rates of SCD or all-cause mortality between patients with a prolonged TpTe compared to a normal interval (Figures 3a and 3b). All-cause mortality in patients with a prolonged TpTe *vs*. normal was 72.3 *vs*. 68.5% (*p* = 0.76). SCD in patients with prolonged TpTe *vs*. normal was 16.0 *vs*. 14.4% (*p* = 0.52).

### OUTCOMES - NORMAL *VS*. PROLONGED TPTE/QT

**Figure 4:**
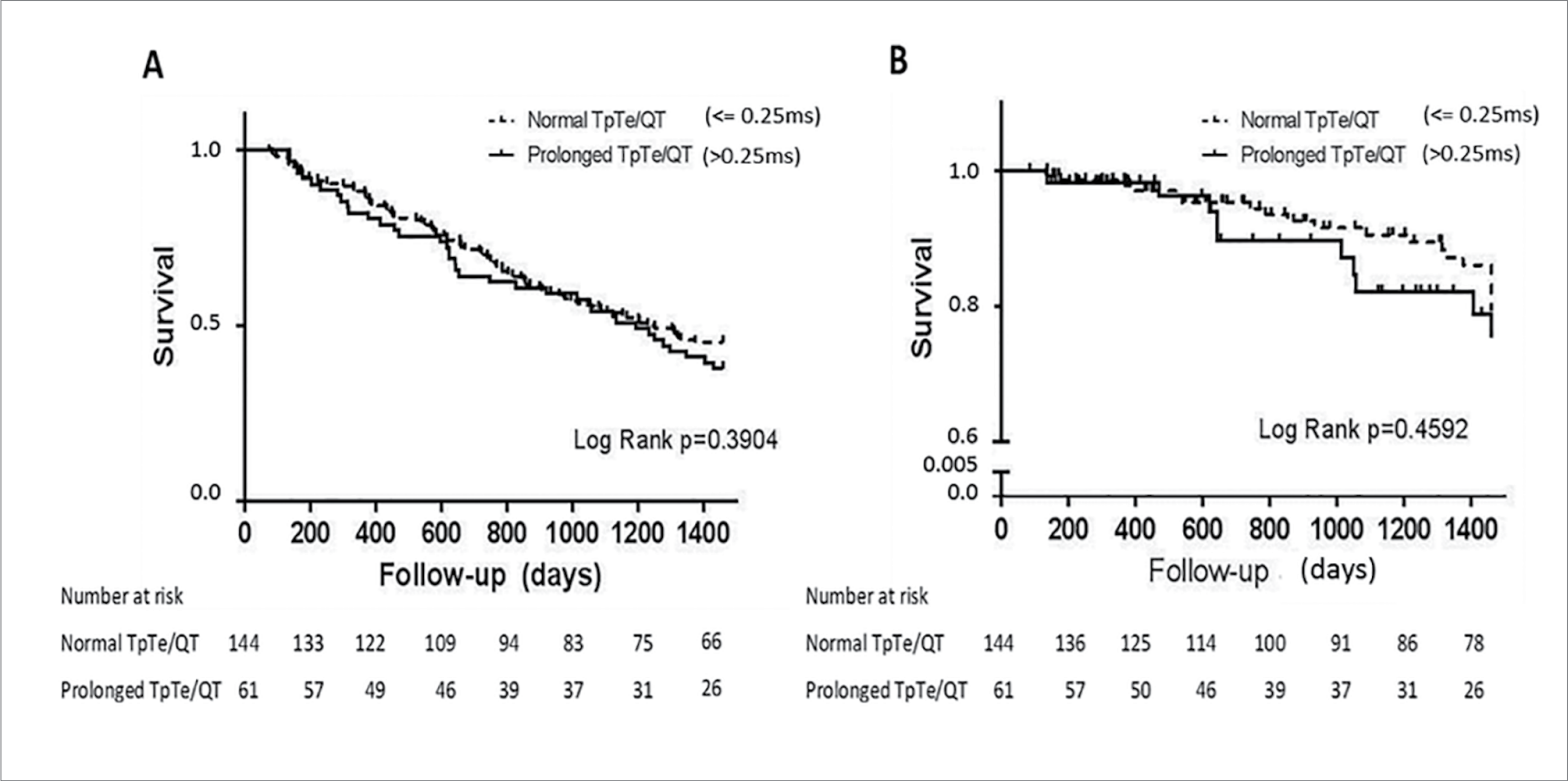
Overall survival (A) and survival without sudden cardiac death (B) between hemodialysis patients with normal and prolonged TpTe/QT interval.

The median survival time for patients with a normal and prolonged TpTe/QT interval was 2.94 and 2.67 years, respectively. Once again, there was no statistically significant difference seen in the rates of SCD or all-cause mortality between the normal and prolonged TpTe/QT groups. All-cause mortality was 68.8 *vs*. 70.8% in patients with prolonged compared to normal TpTe/QT (*p* = 0.26) (Figure 4A). SCD was present in 13.1 *vs*. 16% in patients with prolonged TpTe/QT compared to normal TpTe/QT (*p* = 0.51) (Figure 4B).

### CAUSE OF DEATH

No statistical difference was found in cause of death between patients with normal and prolonged TpTe (Table 2). However, there was a trend of increased mortality from infection in patients with normal TpTe (*p* = 0.09). No significant difference in cause of death was observed when comparing normal and prolonged TpTe/QT groups.

**Table 2:** Causes of death

Cause of Death	All (N = 205)	Normal TpTe (N = 111)	ProlongedTpTe(N = 94)	*p* value (Normal *vs*. Prolonged TpTe)	Normal TpTe/QT (N = 144)	Prolonged TpTe/QT(N = 61)	*p* value (Normal vs. Prolonged TpTe/QT)	*p* value (Prolonged TpTe vs Prolonged TpTe/QT)
SCD, n (%)	31 (15.1)	16 (14.4)	15 (15.9)	0.52	23 (16)	8 (13.1)	0.51	0.94
Infection, n (%)	22 (10.7)	14 (12.6)	8 (8.5)	0.09	18 (12.5)	4 (6.5)	0.88	0.87
Non-SCD cardiac, n (%)	17 (8.2)	10 (9.0)	7 (7.4)	0.28	12 (8.3)	5 (8.1)	0.82	0.65
Unknown, n (%)	18 (8.7)	11 (9.9)	7 (7.4)	0.19	12 (8.3)	6 (9.8)	0.54	0.42
Other, n (%)	56 (27.3)	25 (22.5)	31 (32.9)	0.86	37 (25.6)	19 (31.1)	0.13	0.70
All-cause Mortality, n (%)	144 (70.2)	76 (68.5)	68 (72.3)	0.76	102 (70.8)	42 (68.8)	0.41	0.26

SCD: sudden cardiac death; TpTe: T peak to T end interval; TpTe/QT: TpTe interval corrected for the QT interval.

## DISCUSSION

In this analysis of 205 patients on maintenance hemodialysis therapy, the finding of a prolonged TpTe or prolonged TpTe/QT at or near the time of initiating dialysis was not associated with a statistically significant increase in SCD or all-cause mortality.

It is well established that there is a high incidence of cardiovascular morbidity and mortality in the ESRD population.^1^^,^ 18 Unfortunately, traditional risk factors for cardiovascular (CV) disease, which are ubiquitous among patients with ESRD, provide little predictive discrimination for those at higher risk of developing CV events, especially SCD.^5^^-^ 7 Traditional markers provide general assessments of vascular health and may not be specific enough to influence adequate risk reduction measures in regard to SCD in patients with ESRD. The majority of SCD cases are presumed to be related to ventricular arrhythmias, although the possibility of PEA/asystole or bradycardic arrest is not ruled out in analyses focusing on baseline ECG parameters, as ours. Patients with ESRD, especially those on hemodialysis, are chronically exposed to homeostatic changes that could perturb the electrical conductance of the myocardium. Such changes should readily be evident in a resting ECG but investigating the predictive ability of ECG findings in ESRD patients is not novel. In a sub analysis of the German diabetic dialysis study (4D), 9 ECG parameters were examined for their ability to predict mortality in ESRD patients.^19^ The authors found that the only ECG parameter predictive of increased mortality was the absence of sinus rhythm, while signs of MI, heart rate, QRS axis, AV block, complete LBBB or RBBB, and QT interval had no significant association with outcomes. Several possibilities might explain these findings. First, structural defects are perhaps more important than conduction abnormalities in the genesis of SCD in ESRD. Second, uremia and the subsequent CV changes with the additional stress of the dialysis procedure are the primary determinants of SCD, and thus indifferent to the underlying electrical and structural defects of the heart. Third, routine ECG measurements are not specific enough to the high risk conduction abnormalities in the highly altered environment of ESRD. Our investigation sought to examine this last possibility. We examined the predictive ability of TpTe, an ECG marker of arrhythmogenesis that has been shown to be predictive of SCD in other populations.

It is important to first understand the basis for the TpTe measurement to understand its significance. The TpTe interval represents the speed of the dispersion of the repolarization potential from the epimyocardium to the endomyocardium.^20^^,^ 21 A delay in this interval allows the possibility of pre-excitation and induction of arrhythmia.^22^ This measure has been demonstrated to predict non-sustained ventricular tachycardia post-cardiac resynchronization and ICD firing in patients requiring the placement of Bi-V pacing and ICD, as well as predict overall mortality and VT/VF in patients with systolic dysfunction and ICD implantation for primary prevention.^23^^,^ 24 While a difference in electric potentials (i.e. dispersion) between cell lines will always be present, increases in the dispersion have been linked to worse outcomes in disease states.^14^^-^ 16 ^,^ 25 One explanation as to why TpTe may be prolonged in ESRD patients is the presence of an increase in myocardial fibrosis in this population. Fibrosis can lead to heterogeneous zones of repolarization within the myocardium, which can induce ventricular arrhythmias.^26^

Our study failed to demonstrate a significant association between prolonged TpTe and outcomes in our ESRD study population, despite there being more events numerically (all-cause mortality and SCD) in the prolonged interval groups. Some studies showed improved precision in predicted SDC when TpTe is adjusted for heart rate.^27^ Another possible reason is that the dialysis treatment itself exerts a similar effect on cardiac function and conduction in all patients. In an analysis of the effects of dialysis on TpTe and TpTe/QT, Kalantzi et al. found that both intervals were increased in duration after a single hemodialysis session.^17^ Prolongation of TpTe or TpTe/QT was not associated with changes in electrolytes, which suggest that the changes in TpTe were not related to the large electrolyte shifts that routinely occur in patients receiving hemodialysis. Other authors have examined the effect of hemodialysis on QT dispersion, which is another marker of the dispersion of ventricular repolarization. They also found that QT dispersion was increased after HD sessions.^28^ Additionally, other authors found that high frequency QRS duration was significantly increased after HD sessions, further bolstering the premise that dialysis itself alters ECG parameters.^29^ Nevertheless, such effects should, in theory, have more effect on those with pre-existent abnormalities in the conduction parameters. The question then becomes: are these changes consistent risk parameters over time? It is possible that uremic control over time with dialysis may change these parameters: some patients who started with a prolonged TpTe or TpTe/QT may have improvement over time, or all patients may develop a prolonged TpTe or prolonged TpTe/QT thereby eliminating any potential predictive ability over time. ECG changes over the course of time on hemodialysis or peritoneal dialysis may be more important in the evolving CV mortality related to ESRD. Thus, our study may not have been able to detect a significant effect on mortality without following the TpTe duration throughout many dialysis treatments. Perhaps, the amount by which TpTe changes over time, and not an absolute value, could more accurately predict mortality. Others have suggested that single markers like Tpte will not be effective as a predictive measure as a combination of many simultaneous ECG parameters.^7^^,^ 30

There was a higher proportion of African Americans within the normal TpTe group compared to prolonged TpTe group. Racial differences on standard ECG parameters have been previously shown.^31^ In fact, a recent study as part of the Women's Health Initiative found the upper limit of normal for TpTe to be 10 ms longer in African American women compared to Caucasian, Hispanic, and Asian women.^32^ While that study looked only at women, it seems to contradict our findings that African American men are more likely to have a shorter TpTe interval. Further investigations into the importance of ethnicity and gender on these ECG parameters are needed.

There are several limitations in this study. Due to the way we defined baseline ECG, we cannot rule out a direct effect of hemodialysis duration on TpTe; however, restriction of the population to those who had an ECG within 60 days of initiating dialysis demonstrated similar results with no significant difference in mortality or SCD at 5 years following initiation of hemodialysis (data not shown). We did not collect data on the reason for obtaining the ECG (i.e. routine check *vs*. suspicion for an acute event, e.g. pre-operative clearance *vs*. myocardial infarction); this might have introduced a selection bias. We also were unable to obtain information on medication use at baseline, which is important as certain medications have been shown to affect the TpTe interval. Another limitation is that our observed SCD event rate was lower than what has been previously reported. The SCD rate in the normal TpTe group at 4 years was 14.4% compared to the 19% rate that was used in the *a priori* power analysis and may have led to a loss of power. Another item worth mentioning is that almost 15% of the patients screened were excluded because they did not have an ECG within 18 months of dialysis initiation. Given that these patients are at high risk for adverse cardiac events and likely would benefit from a baseline ECG at dialysis initiation, this should be an area of focus for nephrologists and cardiologists. This also resulted in a relatively small population with limited follow-up time. Lastly, it should be noted that this study represents a Virginia (USA) population that is predominantly male and Caucasian, which limits the generalizability of these findings.

## CONCLUSION

We hypothesized that in an ESRD population the presence of a prolonged TpTe or TpTe/QT segment would be predictive of increased all-cause mortality and/or SCD. Our study was unable to show a statistically significant difference in event rates between groups with prolonged and normal TpTe or TpTe/QT segments.
