# Erratum

**DOI:** 10.1590/2175-8239-JBN-2017-0021er

**Published:** 2020-11-30

In the article “TpTe and TpTe/QT: novel markers to predict sudden cardiac death in ESRD?”, with DOI code number 10.1590/2175-8239-jbn-2017-0021, published at Brazilian Journal of Nephrology, 41(1): 38-47, on the page 38:

Where it was written:

**Abstract**

Introduction: Reliable markers to predict sudden cardiac death (SCD) in patients with end stage renal disease (ESRD) remain elusive, but **echocardiogram** (ECG) parameters may help stratify patients.

**Resumo**

Introdução: Marcadores confiáveis para predizer morte súbita cardíaca (MSC) em pacientes com doença renal terminal (DRT) permanecem elusivos, mas os parâmetros do **ecocardiograma** (ECG) podem ajudar a estratificar os pacientes.

Should read:

**Abstract**

Introduction: Reliable markers to predict sudden cardiac death (SCD) in patients with end stage renal disease (ESRD) remain elusive, but **electrocardiogram** (ECG) parameters may help stratify patients.

**Resumo**

Introdução: Marcadores confiáveis para predizer morte súbita cardíaca (MSC) em pacientes com doença renal terminal (DRT) permanecem elusivos, mas os parâmetros do **eletrocardiograma** (ECG) podem ajudar a estratificar os pacientes.

